# Giant Cell Tumour of the Patella: A Missing Differential Diagnosis in the Young

**DOI:** 10.7759/cureus.25151

**Published:** 2022-05-19

**Authors:** Mashood Iqbal, Abdus Salam Khan, Uzzam Ahmed Khawaja, Amna Saleem

**Affiliations:** 1 Internal Medicine, Jinnah Medical College Hospital, Karachi, PAK; 2 Orthopedic Surgery, Jinnah Medical and Dental College, Karachi, PAK; 3 Internal Medicine, Jinnah Medical and Dental College, Karachi, PAK; 4 Clinical and Translational Research, Larkin Community Hospital, South Miami, USA; 5 Medical School, Jinnah Medical and Dental College, Karachi, PAK

**Keywords:** histopathology, diagnostic radiology, oncology, orthopedics, giant cell tumour of the patella

## Abstract

Herein, we discuss the case of a 26-year-old male patient with a diagnosis of giant cell tumour (GCT) of the patella which is an exceptionally uncommon condition. The motive of reporting a rare case such as the giant cell tumour of the bone (GCTB) relies on its diagnostic incidence. Since the symptoms of this tumour overlap more common etiologies than GCT, the diagnosis of such a devastating malignant tumour is usually missed and hence delayed, which leads to poor treatment strategies and ultimately an irreversible fatal outcome.

## Introduction

The tumors of the patella are rare and more commonly of benign morphology rather than the malignant variant [1]. Statistically, the majority of the patellar tumors comprise 73% of the benign moiety. The benign classification involves giant cell tumors (GCTs) and chondroblastomas, which are mostly found comprising 33% and 16%, respectively [1]. The overall prevalence of giant cell tumor of the bone (GCTB) comprises 4-5% of all primary bone lesions [2]. While the patellar bone involvement is under 1% of all GCTB, GCT is intriguingly considered locally aggressive with an overall predilection towards women than men, and usually occurs in young adults aged 20-45 years [2, 3]. Literature review suggests that GCT most commonly involves the epiphyseal region of the long bones as well as the sacrum or the spine [4].

The motive of reporting a rare case such as GCTB relies on its diagnosing statistics. GCTs are rare, affecting 1 in 1,000,000 people per year, and 50% of those tend to localize around the knee joint [5]. Despite its scarcity and prevalence in knee joints, patients typically present with anterior knee pain, joint swelling, and an exacerbating pain at rest. Due to etiologies more common than GCT, such as the patellar tendinitis, the diagnosis of the former is often delayed [6].

Management criteria of GCT are poorly defined due to its scant diagnosis. However, it includes surgery with curettage or wide excision of the tumor [5]. Ongoing research with targeted therapy with denosumab, which targets the RANK-L molecule, is thought to regulate the growth of GCTs and is often used prior to surgery in order to improve the post-surgical prognosis in the affected individuals [5].

## Case presentation

A 26-year-old male patient, employed at a garment factory, with no known co-morbidities presented to our orthopedic out-patient-department (OPD) with the chief complaint of pain in the left knee of 5 years duration. As per the patient’s history of presenting illness, he was in a usual state of health previously when he initially experienced sudden, throbbing, non-radiating, exertion-related, moderately intense left anterior knee pain which progressively increased with the passage of time. Pain aggravation was witnessed more specifically in routine activities at work and during exercise, partially relieved by over-the-counter analgesics taken as needed. No family history of any infantile or adolescent musculoskeletal disorders was present.

**Figure 1:**
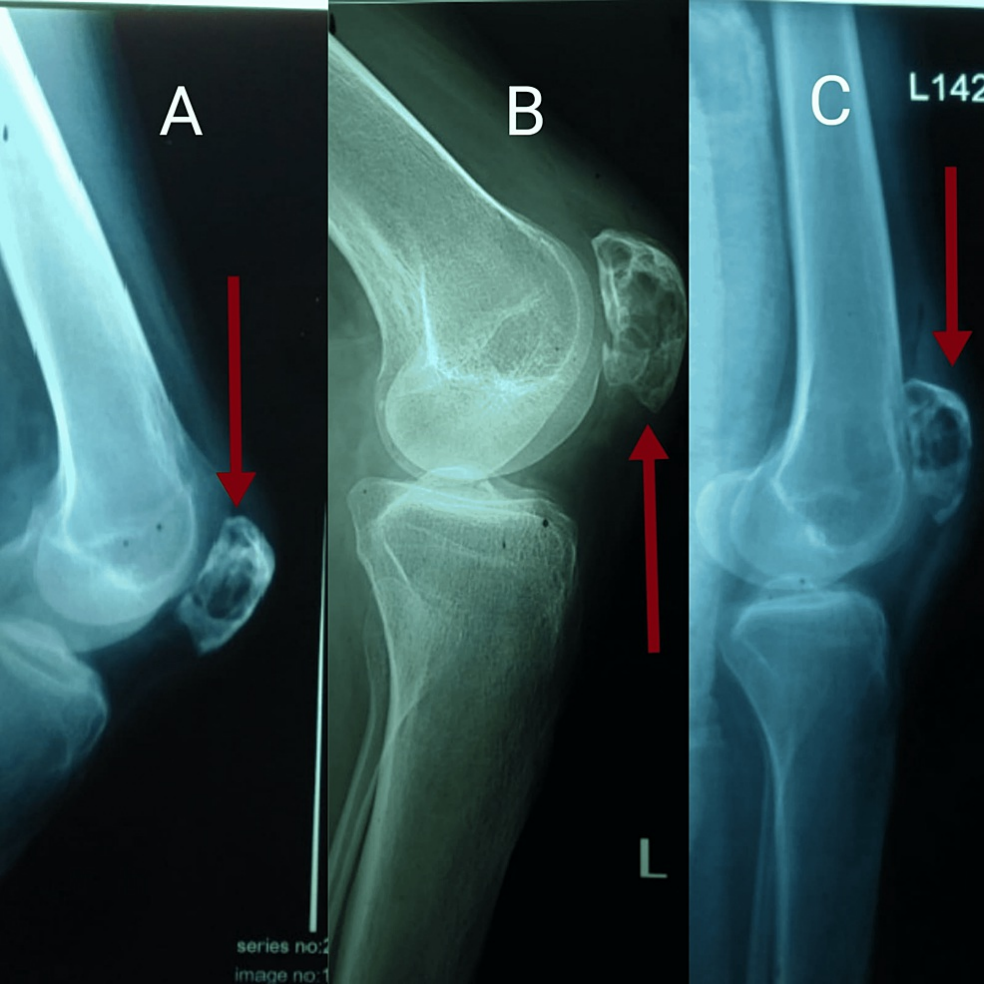
Lateral radiographs of the left knee joint (A-C): well-defined lytic lesions throughout the substance of patellar bone.

**Figure 2:**
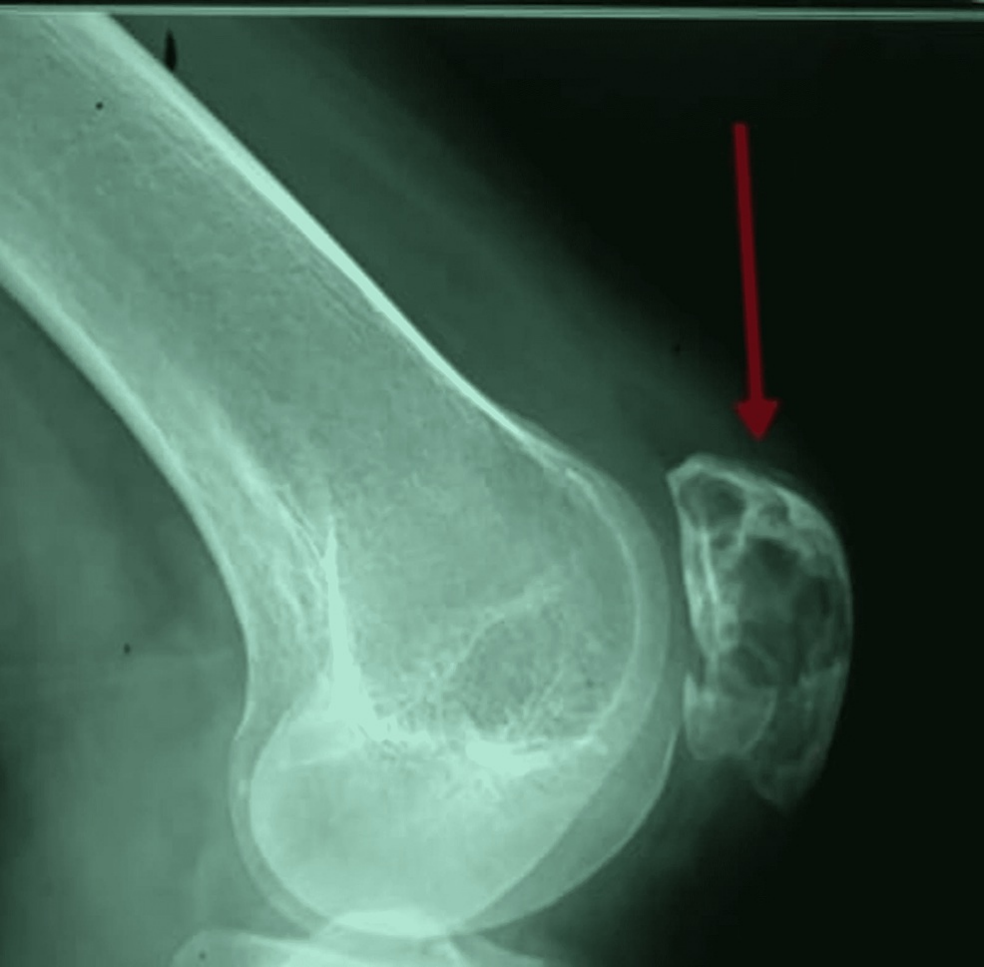
Lateral radiograph of the left knee joint: circumscribed septate osteolytic lesions seen in the patella.

**Figure 3:**
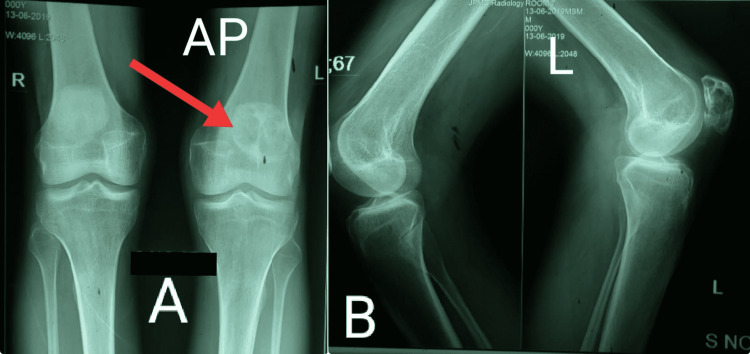
Antero-posterior (A) and lateral bilateral (B) knee joint radiography clearly demarcating the enlarged lytic lesion in the left patellar bone.

**Figure 4:**
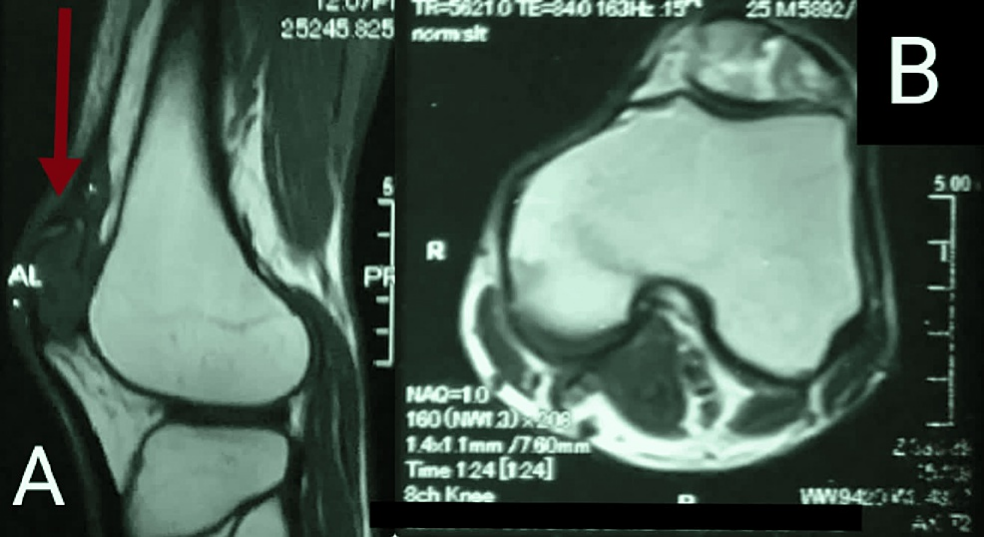
Sagittal (A) and axial (B) MRI slices representing the details of the confined heterogeneous textured patellar lesion. Involvement of the proximal femur and tibia appears negative.

On physical examination of the lower extremities, mild left anterior knee swelling was noted along with size discrepancy between left and right lower extremity, with left lower extremity atrophy. The sensations were intact overall in the affected limb with no skin color changes, discharge, nodules, lymphadenopathy, or alopecia. Powers of the left lower limb were compared with the right and exhibited moderate leg pain during knee flexion and extension against resistance. General physical examination of the skin, nails, and mucous membranes remained unremarkable. Baseline investigations including Complete Blood Count (CBC), Liver Function Tests (LFTs), Urine Detailed Report (D/R), Urea, Creatinine, and Electrolytes (UCE), Chest X-ray (CXR), and viral markers were performed and were found to be unremarkable. To further investigate comprehensively, imaging radiography of the patient was performed including a lateral radiograph of the left knee joint initially (Figure 1A-1C, Figure 2). Bilateral knee joint radiographs were also compared to see the prominent differences (Figure 3). An MRI of the left knee (Figure 4) was obtained which revealed a septate cystic lesion involving the complete substance of the patellar bone.

An active giant cell tumor of the left patellar bone was finally suspected with a differential diagnosis of a solitary benign bone cyst, chondroblastoma, and aneurysmal cystic disease of bone kept into consideration. Biopsy was planned with patellar de-roofing and curettage. Results revealing multi-nucleated giant cells were obtained. The patient could not be followed up further due to his non-compliance to further treatment after the results of his biopsy.

## Discussion

Giant cell tumour, a rare benign neoplasm, accounts for 5% of the primary bone tumours with 40% to 50% of cases reported entailing the knee joint [7, 8]. The knee joint has been clearly elucidated to be involved at a startling rate of 70% according to a case reported by Slachev et al. The tumour presides to its benign configuration in the majority of the cases reported throughout the literature [2]. Due to its rare occurrence, and the involvement of the adolescent and age groups confined between 30 and 40 years, its reporting has sparked the GCT entity to be considered as a differential while evaluating any tenacious knee joint pain relative to flexion and extension against resistance or physical activity [8]. Despite its low incidence and benign nature, the tumour has been found to be locally aggressive [9]. 

Manifestations of the GCT of the patella preside to evolve as knee pain is associated with swelling. Joint erythema, tenderness on palpation, effusion, and crepitus on physical examination have also been classified as a part of the disease symptomatology [10]. The magnitude of symptoms mentioned throughout the studies steadily converges on the localised pain symptoms thereby leading to a casual physician approach of focusing on the patient more specifically with pain management modalities instead of visualising the other aspect of pain due to an uncertain origin [2]. Hence, the latter diagnosis must be considered as a strong differential as part of the evaluation of young patients preferably with persistent knee pain despite consuming on and off pain suppression therapy. Laboratory parameters have been shown to display an increase in the erythrocyte sedimentation rate (ESR) and alkaline phosphatase levels in the serum in affected patients [10].

Imaging techniques have been reported to play a pivotal role in the diagnosis of morbidity [11]. MRI and plain radiographs have been considered fruitful in suspecting the disease which then is ultimately confirmed on biopsy. Initial evaluation with radiography has been proclaimed to have a significant benefit in diagnosing morbidity [11]. Yoshida et al. enlighten the suspicion of the presence of the tumour which was primarily configured by the MRI and radiographic findings hence highlighting the importance of preliminary imaging modality [12]. 

Surgical excision of the tumour has been considered the recommended treatment where intralesional curettage and partial patellectomy have been explained as the two dimensions of the surgical approach [10]. No strict treatment protocol has been well established throughout the literature; however, studies mention the application of patellectomy for aggressive tumours [2]. Malignant tumours involving the patella have been rarely encountered, but may be treated with amputation or radiotherapy in cases where patient compliance to amputation is minimal [13].

## Conclusions

Although patellar tumours are rare, they should always be kept in mind as a differential diagnosis in cases of anterior knee pain and discomfort. It is imperative to note that giant cell tumours of the patella can be difficult to distinguish from other GCTBs on the basis of routine diagnostic imaging and hence warrant histopathological investigation of the specimen obtained post surgery. Surgeons must be careful in mapping the treatment options that heavily rely on the staging of the tumour.
